# 

**Published:** 1893-11

**Authors:** 


### CONTENTS.

ORIGINAL ARTICLES—

Improved Long Traction Hip-Splint, With Proper Method of Applying Adhesive Plasters. By Henry Ling Taylor, M. D., New York ........ 675

Membranous Croup, With Report of Cases Treated With Tracheotomy. By R. W. Harbin, M. D., Calhoun, Ga...... 577

American Orthopedic Association—The Embodiment of an Idea. By A. J. Steele, M. D., St. Louis, Mo.............. 579

Ten Reasons Why Abdominal Bandages Should Not be Used After Labor. By. W. D. Conway, M. D., Athens, Ga....... 580

The Details of the Operation of Circumcision. By J. McF. Gaston, Jr., M. D., Atlanta, Ga........................... 581

Glaucoma. By A. B. Patterson, M. D., Atlanta, Ga.......... 583

CORRESPONDENCE—

A Reply to Dr. J. M. Thomas............................. 587

EDITORIALS—

Cholecystostomy For Gall-Stones, With Occlusion of the Cystic Duct .................................... 614

Philadelphia Medical Society............................. 615

Tri-State Medical Society—Alabama, Georgia and Tennessee 616

Southern Surgical and Gynæcological Association............ 617

Clinical Society of Baltimore............................ 620

Editorial Notes ........................................ 620

Personals ...... .................................... 613

BOOK REVIEWS—
A Text Book of Opthalmogy.................................... 588
Hand Book of Local Therapeutics.............................. 589
A Chapter On Cholera For Lay Readers ......................... 589
Kirk's Hand Book of Physiology............................... 590
Reactions .................................................. 591
A Text Book of Normal Histology.............................. 591
SELECTIONS AND ABSTRACTS—
Hemorrnage From the Umbilicus............................... 586
Incontinence of Urine........................................ 592
A Clinical Lecture on Pneumonia.............................. 596
Long Continued Hemorrhage in the Latter Half of Pregnancy 599
Injuries to the Epiphyses and Their Results.................. 600
Relations of the Vaginal Secretions to Puerperal Fever....... 602
The Treatment of Varicocele................................. 603
Kava-Kava in Gonorrhœa ..................................... 604
The Etiology of Acute Rheumatism............................ 604
Diaphragmatic Pleurisy ..................................... 605
True Croup and Calomel Sublimation ......................... 606
Treatment of Anthrax ........................................ 608
Diagnosis of Inebriety From Appoplexy ...................... 609
Pichi In the Diseases of the Genito-Urinary Tract .......... 610
Crying In Children .......................................... 611
To Detect Tainted Milk....................................... 611
On the Treatment of Empyæmia................................ 612
Treatment of Stricture of the Female Uretha................. 612
Some Results In the Treatment of Epilepsy.................... 613
Papoid In Consumption........................................ 623
Esencia DeCalisaya .......................................... 628
PRESCRIPTION DEPARTMENT—.................................... 622–623
SPECIAL NOTES—.............................................. 624-630

